# Glutathione Reductase Is Associated with the Clinical Outcome of Septic Shock in the Patients Treated Using Continuous Veno-Venous Haemofiltration

**DOI:** 10.3390/medicina57070689

**Published:** 2021-07-06

**Authors:** Georgijs Moisejevs, Eva Bormane, Dace Trumpika, Regina Baufale, Inara Busmane, Julija Voicehovska, Anda Grigane, Olegs Suba, Alise Silova, Andrejs Skesters, Aivars Lejnieks, Linda Gailite, Girts Brigis

**Affiliations:** 1 Department of Internal Diseases, Riga Stradins University, LV-1079 Riga, Latvia; julija.voicehovska@rsu.lv (J.V.); aivars.lejnieks@rsu.lv (A.L.); 2 Clinic of Nephrology and Renal Replacement Therapy, Riga East University Hospital, LV-1079 Riga, Latvia; eva.bormane@aslimnica.lv (E.B.); dace.trumpika@gmail.com (D.T.); rkvele@inbox.lv (R.B.); inara.busmane@gmail.com (I.B.); anda.grigane@aslimnica.lv (A.G.); 3 Clinic of Toxicology and Sepsis, Riga East University Hospital, LV-1079 Riga, Latvia; olegs.suba@aslimnica.lv; 4 Scientific Laboratory of Biochemistry, Riga Stradins University, LV-1067 Riga, Latvia; aliseisilovai@gmail.com (A.S.); andrejs.skesters@rsu.lv (A.S.); 5 Clinic of Internal Diseases, Riga East University Hospital, LV-1079 Riga, Latvia; 6 Scientific Laboratory of Molecular Genetics, Riga Stradins Univerisy, LV-1007 Riga, Latvia; linda.gailite@rsu.lv; 7 Department of Public Health and Epidemiology, Riga Stradins Univerisy, LV-1010 Riga, Latvia; girts.brigis@rsu.lv

**Keywords:** glutathione reductase, oxidative stress, septic shock, continuous veno-venous haemofiltration

## Abstract

*Background and objectives:* At present, there is insufficient evidence to support the use of continuous veno-venous haemofiltration (CVVH) in the early treatment of septic shock. This study focuses on the association between survival and different parameters of oxidative stress (RedOx). Thereby, we evaluated whether RedOx markers are associated with the outcome of septic shock in patients under early-initiated CVVH treatment. *Materials and Methods:* We conducted a prospective observational study of 65 patients with septic shock who started CVVH within 12 h after hospital admission. Blood samples were taken from each patient prior to the start of CVVH. The following RedOx markers were measured: glutathione peroxidase, glutathione reductase (GR), total antioxidant capacity, superoxide dismutase, nitric oxide, malondialdehyde and 4-hydroxynonenal. The odds ratio (OR) was calculated using binary logistic regression and stepwise multivariable regression. *Results:* The 65 patients had a median age of 66 years and 39 were male. Based on the outcome, the patients were divided into two groups—non-survivors (*n* = 29) and survivors (*n* = 36)—and the levels of RedOx markers were compared between them. Of all the markers, only higher GR activity was found to be significantly associated with the fatal outcome; 100.3 U/L versus 60.5 U/L, OR = 1.027 (95% CI, 1.010–1.044). Following adjustment for the sequential organ failure assessment score and other parameters, GR activity still presented a significant association with the fatal outcome, OR = 1.020 (95% CI, 1.002–1.038). *Conclusions:* GR activity is associated with in-hospital fatal outcomes among septic shock patients under early-initiated CVVH treatment. Septic shock patients who have a lower GR activity at hospital admission may have a favourable outcome of the early initiation of CVVH.

## 1. Introduction

Sepsis is defined as a life-threatening organ dysfunction syndrome driven by a dysregulated host response to infection. Staggeringly, septic shock—which can develop during sepsis—increases hospital mortality by 40% [1]. Extracorporeal detoxification methods such as continuous veno-venous haemofiltration (CVVH) can remove a number of inflammatory mediators and toxic metabolic waste products [2,3]. Nevertheless, the latest international guidelines for the management of sepsis and septic shock published in 2016 make no supportive recommendation regarding the use of CVVH due to insufficient strong evidence [4]. Data from seven clinical trials have shown that CVVH treatment reduces short-term and long-term mortality in septic patients [5]. However, a meta-analysis of only three clinical trials reported no significant differences in survival between septic patients on conservative treatment and those on treatment combined with high-volume haemofiltration [6]. Currently, none of the extracorporeal detoxification therapy methods is considered to be the most advisable one for septic patients [7], but CVVH is the one most frequently used. Due to the heterogeneous nature of sepsis, septic shock patients who could benefit from early-initiated CVVH treatment may be able to be identified by stratifying them according to different markers, such as inflammatory agents, cytokines and oxidative stress parameters.

Oxidative stress is the imbalance between the production of reactive oxygen species and the endogenous antioxidant defence system [8,9,10]. The glutathione (GSH) defence system is maintained by the glutathione peroxidase (GPx) and glutathione reductase (GR) enzymes. GPx scavenges hydrogen peroxide, and the oxidised form of GSH (GSSG) is formed. Subsequently, GSSG is reconverted to GSH by GR [9]. Another antioxidant defence enzyme is superoxide dismutase (SOD), which catalyses the dismutation of superoxide radicals to hydrogen peroxide and oxygen [10]. Overexpression of nitric oxide (NO) during sepsis leads to decreased peripheral vascular resistance, neutrophil migration failure and formation of peroxynitrite radicals in a reaction with superoxide anions [11]. During oxidative stress, malondialdehyde (MDA) and 4-hydroxynonenal (HNE) are formed as a result of lipid peroxidation. HNE is formed during lipid peroxidation of arachidonic acid (ARA), and lipopolysaccharides have an ability to increase the release of ARA [12]. Thereafter, MDA and HNE react with proteins and form products with direct cytotoxic and genotoxic effects [12,13]. Furthermore, it has previously been shown that the total antioxidant capacity (TAC) is associated with outcome in septic patients [14,15,16]. Thus, the aim of this study was to investigate whether there are any associations between oxidative stress markers and survival in septic shock patients under early-initiated CVVH treatment.

## 2. Materials and Methods

### 2.1. Patients

Our prospective single-centre observational study was conducted at the Clinic of Toxicology and Sepsis, Riga East University Hospital, Latvia, between January 2019 and August 2020. The study comprised patients with the diagnosis of septic shock in accordance with the Third International Consensus Definitions for Sepsis and Septic Shock (Sepsis-3) [1]. The exclusion criteria were pregnancy and age under 18 years. All the patients were treated in accordance with [4]. Within 12 h of diagnosis, all the septic shock patients started CVVH.

All the patients were treated with postdilutional CVVH with various potassium concentrations adjusted to the patient’s needs. For anticoagulation, continuous infusion of unfractionated heparin was used in all the patients without contraindications for anticoagulants; dose estimation was based on the activated clotting time in accordance with the local hospital protocol. Prior to CVVH administration, a venous blood sample from the central venous catheter was drawn from each patient.

At the time of hospital admission, demographic and clinical data were collected from each patient. The sequential organ failure assessment (SOFA) score [1] and the Acute Physiology And Chronic Health Evaluation II (APACHE II) score [17] were calculated on the basis of the collected data. The primary outcome was defined as in-hospital mortality, and all the patients were divided into two groups: survivors and non-survivors.

### 2.2. Laboratory Methods

Following collection, the sample of whole blood was immediately stored at −20 °C for the measurement of GPx. For SOD measurement, red blood cells (RBC) were washed out of the sample of whole blood and stored at −20 °C. The remainder of the whole blood sample was centrifuged at 3000 rpm for 10 min at 4 °C and the resultant plasma was stored at −80 °C.

Whole blood GPx activity, RBC lysate SOD activity, plasma GR activity and plasma TAC concentration were measured using commercial assay kits (Randox Laboratories Ltd., Crumlin, UK) on an automated analyser (RX Daytona™; Randox Laboratories Ltd., Crumlin, UK). Plasma concentrations of MDA and HNE adducts were measured using an OxiSelect™ TBARS Assay Kit and an HNE Adduct Competitive ELISA Kit (Cell Biolabs Inc., San Diego, CA, USA), respectively, following the manufacturers’ instructions. A SPARK™ multimode microplate reader (Tecan, Grödig, Austria) was used to read the plates. Plasma NO concentration was measured using a QuantiChrom™ Nitric Oxide Assay Kit (BioAssay Systems, Hayward, CA, USA) according to the manufacturer’s instructions, and the plates were read on a Sunrise™ absorbance microplate reader (Tecan).

### 2.3. Statistical Analysis

All the continuous variables are shown as the medians and interquartile ranges (IQR). Associations of all the categorical and continuous independent variables with the outcome were assessed by binary logistic regression analysis and odds ratio (OR) together with the 95% confidence intervals (95% CI) were calculated. To investigate which variables were independently associated with the fatal outcome during hospital stay, multivariable analysis using a stepwise logistic regression model (with an entry level of 0.05 and a stay level of 0.05) was applied to the variables which exhibited statistical differences between the survivors and non-survivors. Furthermore, to investigate the discriminative ability of the markers to predict the clinical outcome, receiver operating characteristic analysis was conducted and the area under the curve (AUC) was calculated. All the statistical analyses were performed using SPSS version 23.0 for Mac (SPSS, North Castle, NY, USA).

### 2.4. Ethical Considerations

All the patients or their legal representatives gave signed informed consent and the study protocol was approved by the Central Medical Ethics Committee of Latvia (No. 2/18-06-07 (07.06.2018)).

## 3. Results

### 3.1. Patient Characteristics

During the study period, 65 patients were enrolled. Of these patients, 36 (55.4%) were survivors and 29 (44.6%) were non-survivors. The median length of time on CVVH treatment among the survivors was 51 h (IQR, 37–67), whereas among the non-survivors it was only 35 h (IQR, 13–51) (*p* = 0.035) due to patient mortality. There was no difference in the dose of CVVH treatment between the two groups of patients; the median substitutional fluid flow rate in the survivors was 22.4 mL/kg/h (IQR, 20.6–25.6) and 23.3 mL/kg/h (IQR, 20.7–26.0) in the non-survivors (*p* = 0.379).

The median length of hospital stay of the survivors was 20 days (IQR, 13–33), whereas it was 4 days (IQR, 2–13) for the non-survivors (*p* = 0.004). Furthermore, the median length of stay in the ICU was 8 days (IQR, 6–18) for the survivors and 3 days (IQR, 2–10) for the non-survivors (*p* = 0.292). The SOFA and APACHE II scores were significantly different between the two patient groups (*p* = 0.001 for both scores), with higher scores in the non-survivor group (Table 1).

### 3.2. Blood Chemistry Parameters

The concentration of serum lactate was significantly higher in the non-survivor group than in the survivor group; median values of 4.7 mmol/L (IQR, 3.5–9.4) and 2.8 mmol/L (IQR, 2.1–3.9), respectively (*p* = 0.036). However, there were no significant differences between the two patient groups regarding the concentrations of inflammatory markers C-reactive protein and procalcitonin, as well as the estimated glomerular filtration rate (MDRD equation).

**Figure 1:**
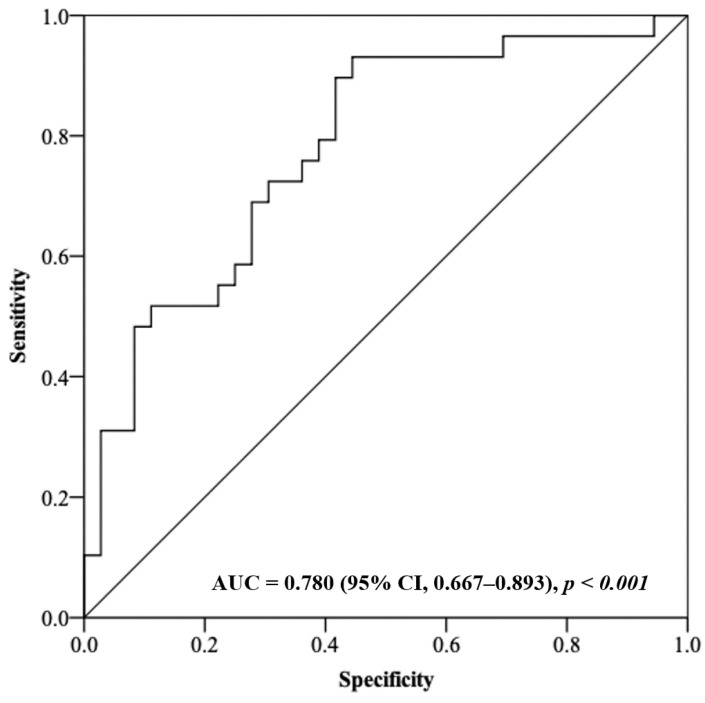
Receiver operating characteristic analysis using plasma GR activity as a predictor of the clinical outcome in septic shock patients.

The non-survivors had a significantly higher plasma GR activity in comparison to the survivors (*p* = 0.002); 100.3 U/L (IQR, 71.8–149.8) versus 60.5 U/L (IQR, 45.0–93.4), respectively, OR = 1.027 (95% CI, 1.010–1.044). All the other oxidative stress/antioxidant markers were not significantly different between the two groups (Table 2). Receiver operating characteristic analysis was subsequently conducted to determine whether GR activity could be used to predict the clinical outcome in septic shock patients treated with CVVH; the AUC to predict the fatal outcome was 0.780 (95% CI, 0.667–0.893) (Figure 1).

### 3.3. Multivariable Analysis

The multivariable analysis using a stepwise logistic regression model included all the parameters presenting a significant difference in the univariable analysis. The increase in the SOFA score (OR = 1.371; 95% CI, 1.032–1.820) and the increase in plasma GR activity (OR = 1.020; 95% CI, 1.002–1.038) were significantly associated with the fatal outcome during hospital stay. Surgical intervention and corticosteroid administration included in the model after adjustment exhibited no significant association (Table 3).

## 4. Discussion

This is the first study to investigate the role of oxidative markers in the prognosis of survival of septic shock patients treated with early-initiated CVVH. Until now, the majority of the studies analysing sepsis and oxidative stress have examined only a small number of patients treated with CVVH.

We found that the GR activity in plasma was significantly higher among the non-survivors in comparison to the survivors of septic shock. Our data are in contrast to those of a similar study published in the literature which reported an association between the lower GR activity in plasma/erythrocytes and the fatal outcome in 50 septic shock patients [18]. It has been proposed that GR activity should be considered as a GSH/GSSG activity estimator [19], consistently with the findings of Kim et al. [18]. On the other hand, the data published by Karapetsa’s group showed no significant difference in GSH/GSSG between sepsis survivors and non-survivors when measured on the first day after diagnosis [20]. However, the data from an animal study showed a higher GR activity in the liver of rats infected with Gram-negative bacteria as well as a remarkable increase of glutathione synthesis in organs [21]. Another animal sepsis model demonstrated a decreased GR activity in the liver and lung tissue every three hours after sepsis onset [22]. Based on the information available, we proffer that high GR activity among septic patients on the first day of sepsis onset represents a hyperinflammatory response which predicts the future outcome.

Our data demonstrated no significant difference in GPx activity between the survivors and the non-survivors. This is in line with two previous studies examining 100 and 110 patients [23,24]. However, other studies showed a higher GPx activity among septic shock survivors [18,25]. The data from experimental animal studies showed a higher GPx activity in septic animals [21,22]. Nevertheless, despite all these disparities, the GSH system is one of the most important antioxidant systems involved in the early response to oxidative damage [9,26].

We found that TAC presented a high OR in the non-survivor group without a significant difference. This finding was also reported in two previous studies [20,27]. However, other studies reported that a high TAC concentration is a strong predictor of septic patient mortality [14,15,16]. These studies measured the TAC concentration in serum, whereas we measured the TAC concentration in plasma. Even so, the role of the TAC in the pathogenesis of sepsis is unclear, although it may be that a high TAC concentration could be due to the increase of antioxidative activity to compensate proinflammatory mechanisms in the ebb phase of septic shock.

Our data demonstrated a higher MDA concentration in the non-survivors; however, the difference between the survivors and the non-survivors did not reach statistical significance. Several previous studies showed significantly higher MDA concentrations in non-survivors of sepsis and septic shock [14,15,18,25,27]. This discrepancy may be due to different patient numbers and/or different blood collection times. Conversely to MDA, our data demonstrated a lower HNE adduct concentration in the non-survivors without showing a significant difference. This lower concentration may be due to the very reactive nature of HNE which, under experimental conditions after exposure to GSH, is fully consumed within one hour, in contrast to MDA [12,13]. At present, there are no studies on the concentration of HNE adducts in sepsis patients in the literature. However, studies using animal models of sepsis showed an increased HNE concentration in blood or overexpression in organ tissues after sepsis initiation [26,28,29].

We found no difference in SOD activity between the two patient groups. However, a study by Costa et al. demonstrated a higher SOD activity in septic shock survivors only after adjustment for age and protein carbonyls [30]. This adjustment for protein carbonyls may explain the discrepancy with our data. In contrast, a study by Warner et al. reported a higher SOD concentration in non-survivors of sepsis [31]. That study examined only 32 patients and measured the SOD concentration instead of the activity. Thus, SOD could be a helpful marker in the evaluation of septic shock patients, but only if analysed in combination with other markers, such as protein carbonyls.

Our data demonstrated a higher NO concentration in the non-survivors without showing a significant difference. This finding is in accordance with the work of Yu et al. [32]. A study by Winkler et al. demonstrated changes in the NO metabolism during sepsis, and it was suggested that decreased NO synthesis may mimic impairment of the immune response mechanism [33].

The main limitations of the present study are its relatively small number of patients, absence of repetitive measurements during the clinical course and patient recruitment from only one study centre. Another significant limitation in common with most studies of this type is the heterogeneity of the underlying pathologies that lead to sepsis development.

## 5. Conclusions

This study demonstrates that plasma GR activity is associated with in-hospital fatal outcome of septic shock patients under early-initiated CVVH treatment. Septic shock patients who have a lower GR activity at hospital admission may have a favourable outcome of the early initiation of CVVH. Future studies should focus on investigating further the role of the GSH system markers in septic shock patients, including the concurrent measurement of multiple markers and repetitive measurements during the course of the disease.

## Figures and Tables

**Table 1:** Univariable comparison of general characteristics between the two patient groups.

Characteristics	Survivors (*n* = 36)	Non-Survivors (*n* = 29)	OR	95% CI	*p*-Value
Age, years	63 (50–77)	67 (58–76)	1.012	0.978–1.047	0.497
Male gender, *n* (%)	24 (66.7%)	15 (51.7%)	0.536	0.196–1.464	0.224
Comorbidities, *n* (%)					
Cardiovascular disease	20 (55.6%)	18 (62.1%)	1.309	0.483–3.549	0.597
Diabetes mellitus	13 (36.1%)	9 (31.0%)	0.796	0.281–2.252	0.667
Neoplasia	3 (8.3%)	6 (20.7%)	2.870	0.650–12.664	0.164
Site of infection, *n* (%)					
Respiratory	7 (19.4%)	14 (48.3%)	2.000	0.108–36.954	0.641
Urogenital	9 (25.0%)	7 (24.1%)	0.778	0.041–14.750	0.867
Biliary	3 (8.3%)	0 (0.0%)	-	-	-
Soft tissue	5 (13.9%)	2 (6.9%)	0.400	0.016–10.017	0.577
Intraabdominal	11 (30.6%)	5 (17.2%)	0.455	0.023–8.829	0.602
Other	1 (2.8%)	1 (3.4%)	1.000	-	-
SOFA score	10 (9–12)	12 (12–16)	1.584	1.216–2.062	0.001
APACHE II score	19.5 (16–25)	27.0 (25–31)	1.184	1.075–1.305	0.001
Management, *n* (%)					
Surgical intervention	18 (50.0%)	6 (20.7%)	0.261	0.086–0.792	0.018
Mechanical lung ventilation	22 (61.1%)	24 (82.8%)	3.055	0.945–9.877	0.062
Corticosteroids	24 (66.7%)	26 (89.7%)	4.333	1.089–17.250	0.037

**Table 2:** Univariable comparison of blood chemistry parameters between the two patient groups.

Parameters	Survivors (*n* = 36)	Non-Survivors (*n* = 29)	OR	95% CI	*p*-Value
Lactate, mmol/L	2.8 (2.1–3.9)	4.7 (3.5–9.4)	1.184	1.011–1.385	0.036
Creatinine, mcmol/L	203 (148–348)	271 (169–480)	1.003	1.000–1.005	0.047
eGFR (MDRD)	25.6 (15.9–44.4)	16.6 (9.45–32.3)	0.985	0.964–1.007	0.172
C-reactive protein, mg/L	269 (138–356)	268 (195–429)	1.001	0.998–1.004	0.486
Procalcitonin, ng/mL	25.8 (7.2–121.1)	51.8 (18.3–132.8)	0.999	0.995–1.003	0.702
Total antioxidant capacity, mmol/L	1.84 (1.67–2.20)	1.92 (1.67–2.61)	2.124	0.914–4.936	0.080
Glutathione peroxidase, U/L	8733 (7247–13,091)	10709 (7770–12,561)	1.000	1.000–1.000	0.445
Superoxide dismutase, U/gHb	1764 (1700–1946)	1810 (1640–2156)	1.001	0.999–1.002	0.448
Nitric oxide, mcM	31.0 (18.2–50.0)	36.6 (21.1–77.2)	1.014	0.996–1.033	0.119
Malondialdehyde, mcM	6.16 (4.66–8.47)	7.40 (4.17–13.40)	1.016	0.964–1.072	0.567
4-Hydroxynonenal adducts, mcM	1.16 (0.00–4.94)	0.01 (0.00–1.84)	0.862	0.730–1.018	0.079
Glutathione reductase, U/L	60.5 (45.0–93.4)	100.3 (71.8–149.8)	1.027	1.010–1.044	0.002

**Table 3:** Multivariable analysis of the factors significantly associated with in-hospital survival of septic shock patients.

Parameters	Univariable Analysis			Multivariable Analysis		
	OR	95% CI	*p*-Value	OR	95% CI	*p*-Value
Management						
Surgical intervention	0.261	0.086–0.792	0.018	0.485	0.126–1.871	0.293
Corticosteroids	4.333	1.089–17.250	0.037	2.855	0.588–13.866	0.193
SOFA score	1.584	1.216–2.062	0.001	1.371	1.032–1.820	0.029
APACHE II score	1.184	1.075–1.305	0.001	-	-	-
Lactate, mmol/L	1.184	1.011–1.385	0.036	-	-	-
Glutathione reductase, U/L	1.027	1.010–1.044	0.002	1.020	1.002–1.038	0.028

## References

[1] Singer M., Deutschman C.S., Seymour C.W., Shankar-Hari M., Annane D., Bauer M., Bellomo R., Bernard G.R., Chiche J.D., Coopersmith C.M. (2016). The Third International Consensus Definitions for Sepsis and Septic Shock (Sepsis-3). JAMA.

[2] Rimmelé T., Kellum J.A. (2011). Clinical review: Blood purification for sepsis. Crit. Care.

[3] Schefold J.C., Hasper D., Jörres A. (2009). Organ crosstalk in critically ill patients: Hemofiltration and immunomodulation in sepsis. Blood Purif..

[4] Rhodes A., Evans L.E., Alhazzani W., Levy M.M., Antonelli M., Ferrer R., Kumar A., Servansky J.E., Sprung C.L., Nunnally M.E. (2017). Surviving Sepsis Campaign: International Guidelines for Management of Sepsis and Septic Shock: 2016. Intensive Care Med..

[5] Putzu A., Fang M.X., Boscolo Berto M., Belletti A., Cabrini L., Cassina T., Landoni G. (2017). Blood purification with continuous veno-venous hemofiltration in patients with sepsis or ARDS: A systematic review and meta-analysis. Minerva Anestesiol..

[6] Yin F., Zhang F., Liu S., Ning B. (2020). The therapeutic effect of high-volume hemofiltration on sepsis: A systematic review and meta-analysis. Ann. Transl. Med..

[7] Zha J., Li C., Cheng G., Huang L., Bai Z., Fang C. (2019). The efficacy of renal replacement therapy strategies for septic-acute kidney injury: A PRISMA-compliant network meta-analysis. Medicine.

[8] Bar-Or D., Carrick M.M., Mains C.W., Rael L.T., Slone D., Brody E.N. (2015). Sepsis, oxidative stress, and hypoxia: Are there clues to better treatment?. Redox Rep..

[9] Biolo G., Antonione R., De Cicco M. (2007). Glutathione metabolism in sepsis. Crit. Care Med..

[10] Ritter C., Andrades M., Frota Júnior M.L., Bonatto F., Pinho R.A., Polydoro M., Klamt F., Pinheiro C.T.S., Menna-Barreto S.S., Moreira J.C. (2003). Oxidative parameters and mortality in sepsis induced by cecal ligation and perforation. Intensive Care Med..

[11] Spiller F., Oliveira Formiga R., Fernandes da Silva Coimbra J., Alves-Filho J.C., Cunha T.M., Cunha F.Q. (2019). Targeting nitric oxide as a key modulator of sepsis, arthritis and pain. Nitric Oxide.

[12] Yang B., Fritsche K.L., Beversdorf D.Q., Zezong G., Lee J.C., Folk W.R., Greenlief C.M., Sun G.Y. (2019). Yin-yang mechanisms regulating lipid peroxidation of docosahexaenoic acid and arachidonic acid in the central nervous system. Front. Neurol..

[13] Esterbauer H., Schaur R.J., Zollner H. (1991). Chemistry and biochemistry of 4-hydroxynonenal, malonaldehyde and related aldehydes. Free Radic Biol. Med..

[14] Lorente L., Martín M.M., Pérez-Cejas A., Abreu-González P., López R.O., Ferreres J., Solé-Violán J., Labarta L., Díaz C., Palmero S. (2018). Serum total antioxidant capacity during the first week of sepsis and mortality. J. Crit. Care.

[15] Lorente L., Martín M.M., Almeida T., Abreu-González P., Ferreres J., Solé-Violán J., Labarta L., Jiménez A. (2015). Association between serum total antioxidant capacity and mortality in severe septic patients. J. Crit. Care.

[16] Chuang C.C., Shiesh S.C., Chi C.H., Tu Y.F., Hor L.I., Shieh C.C., Chen M.F. (2006). Serum total antioxidant capacity reflects severity of illness in patients with severe sepsis. Crit. Care.

[17] Knaus W.A., Draper E.A., Wagner D.P., Zimmerman J.E. (1985). APACHE II: A severity of disease classification system. Crit. Care Med..

[18] Kim J.S., Kwon W.Y., Suh G.J., Kim K.S., Jung Y.S., Kim S.H., Lee S.E. (2016). Plasma glutathione reductase activity and prognosis of septic shock. J. Surg. Res..

[19] Yan J., Ralston M.M., Meng X., Bongiovanni K.D., Jones A.L., Benndorf R., Nelin L.D., Frazier W.J., Rogers L.K., Smith C.V. (2013). Glutathione reductase is essential for host defense against bacterial infection. Free Radic Biol. Med..

[20] Karapetsa M., Pitsika M., Goutzourelas N., Stagos D., Tousia Becker A., Zakynthinos E. (2013). Oxidative status in ICU patients with septic shock. Food Chem. Toxicol..

[21] Malmezat T., Breuillé D., Capitan P., Mirand P.P., Obled C. (2000). Glutathione turnover is increased during the acute phase of sepsis in rats. J. Nutr..

[22] Hansson L., Seidegård J., Johansson L., Jeppsson B. (2000). Influence of glutathione metabolising enzymes in rats with gram-negative sepsis. Eur. J. Surg..

[23] Costa N.A., Gut A.L., Pimentel J.A., Cozzolino S.M., Azevedo P.S., Fernandes A.A., Polegato B.F., Tanni S.E., Gaiolla R.D., Zornoff L.A.M. (2014). Erythrocyte selenium concentration predicts intensive care unit and hospital mortality in patients with septic shock: A prospective observational study. Crit. Care.

[24] Hsiao S.Y., Kung C.T., Su C.M., Lai Y.R., Huang C.C., Tsai N.W., Wang H.C., Cheng B.C., Su Y.J., Lin W.C. (2020). Impact of oxidative stress on treatment outcomes in adult patients with sepsis: A prospective study. Medicine.

[25] Ogilvie A.C., Groeneveld A.B., Straub J.P., Thijs L.G. (1991). Plasma lipid peroxides and antioxidants in human septic shock. Intensive Care Med..

[26] Carbonell L.F., Nadal J.A., Llanos M.C., Hernández I., Nava E., Díaz J. (2000). Depletion of liver glutathione potentiates the oxidative stress and decreases nitric oxide synthesis in a rat endotoxin shock model. Crit. Care Med..

[27] Tsai K., Hsu T., Kong C., Lin K., Lu F. (2000). Is the endogenous peroxyl-radical scavenging capacity of plasma protective in systemic inflammatory disorders in humans?. Free Radic Biol. Med..

[28] Song Y., Miao S., Li Y., Fu H. (2019). Ulinastatin attenuates liver injury and inflammation in a cecal ligation and puncture induced sepsis mouse model. J. Cell Biochem..

[29] Kim J.Y., Leem J., Hong H.L. (2020). Protective Effects of SPA0355, a Thiourea Analogue, Against Lipopolysaccharide-Induced Acute Kidney Injury in Mice. Antioxidants.

[30] Costa N.A., Cunha N.B., Gut A.L., Azevedo P.S., Polegato B.F., Zornoff L.A.M., de Pavia S.A.R., Reis B.Z., Fernandes A.A.H., Rogero M.M. (2018). Erythrocyte SOD1 activity, but not SOD1 polymorphisms, is associated with ICU mortality in patients with septic shock. Free Radic Biol. Med..

[31] Warner A., Bencosme A., Healy D., Verme C. (1995). Prognostic role of antioxidant enzymes in sepsis: Preliminary assessment. Clin. Chem..

[32] Yu M.H., Chen M.H., Han F., Li Q., Sun R.H., Tu Y.X. (2018). Prognostic value of the biomarkers serum amyloid A and nitric oxide in patients with sepsis. Int. Immunopharmacol..

[33] Winkler M.S., Kluge S., Holzmann M., Moritz E., Robbe L., Bauer A., Zahrte C., Priefler M., Schwedhelm E., Boger R.H. (2017). Markers of nitric oxide are associated with sepsis severity: An observational study. Crit. Care.

